# Impaired Regulation of Histone Methylation and Acetylation Underlies Specific Neurodevelopmental Disorders

**DOI:** 10.3389/fgene.2020.613098

**Published:** 2021-01-08

**Authors:** Merrick S. Fallah, Dora Szarics, Clara M. Robson, James H. Eubanks

**Affiliations:** ^1^Division of Experimental and Translational Neuroscience, Krembil Research Institute, University Health Network, Toronto, ON, Canada; ^2^Department of Pharmacology and Toxicology, University of Toronto, Toronto, ON, Canada; ^3^Department of Surgery (Neurosurgery), University of Toronto, Toronto, ON, Canada; ^4^Institute of Medical Science, University of Toronto, Toronto, ON, Canada; ^5^Department of Physiology, University of Toronto, Toronto, ON, Canada

**Keywords:** epigenetics, histone, acetylation, methylation, neurodevelopment

## Abstract

Epigenetic processes are critical for governing the complex spatiotemporal patterns of gene expression in neurodevelopment. One such mechanism is the dynamic network of post-translational histone modifications that facilitate recruitment of transcription factors or even directly alter chromatin structure to modulate gene expression. This is a tightly regulated system, and mutations affecting the function of a single histone-modifying enzyme can shift the normal epigenetic balance and cause detrimental developmental consequences. In this review, we will examine select neurodevelopmental conditions that arise from mutations in genes encoding enzymes that regulate histone methylation and acetylation. The methylation-related conditions discussed include Wiedemann-Steiner, Kabuki, and Sotos syndromes, and the acetylation-related conditions include Rubinstein-Taybi, KAT6A, genitopatellar/Say-Barber-Biesecker-Young-Simpson, and brachydactyly mental retardation syndromes. In particular, we will discuss the clinical/phenotypic and genetic basis of these conditions and the model systems that have been developed to better elucidate cellular and systemic pathological mechanisms.

## Introduction

The “neurodevelopmental disorders” represent a group of conditions in which altered brain development leads to cognitive, neurological, and/or psychiatric impairments in children (Thapar et al., 2017). While multiple causes exist, this broad group of disorders houses a number of genetic conditions, in which spontaneous or inherited genetic variations are the specific cause for the neurodevelopmental phenotypes (Niemi et al., 2018). Of these, there are mutations, including histone modifiers, that affect epigenetic-related cell machinery (Millan, 2013).

An early definition of epigenetics by Russo et al. states that it is “the study of mitotically and/or meiotically heritable changes in gene function that cannot be explained by changes in DNA sequence” (Russo et al., 1996). This broad definition was later refined as “the structural adaptation of chromosomal regions so as to register, signal or perpetuate altered activity states” (Bird, 2007). These modifications can be inherited or may develop during the lifetime (Morgan et al., 1999; Daxinger and Whitelaw, 2012). Epigenetic modifications are thought to involve multiple processes, including an epigenator signal, an initiator, and a maintainer (Berger et al., 2009). Briefly, epigenetic modifications often begin when an extracellular signal, or epigenator, interacts with a host cell. This transient event (for example, a post-translational protein modification) activates the epigenetic initiator. This initiator (for example, a DNA binding protein or non-coding RNA) in turn facilitates a location specific modification of chromatin structure. The epigenetic maintainer (for example, histone modifiers or DNA methylators), which may have been recruited by the initiator, then stabilizes the epigenetic signal to maintain the chromatin modification (Berger et al., 2009).

Histone modifications are one example of epigenetic alteration, and represent a key mechanism through which local gene expression is regulated (Cedar and Bergman, 2009). Histones play a major role in determining chromatin structure. The vast majority of genomic DNA is found associated with nucleosomes; octameric groups of histone proteins comprised largely of H2A, H2B, H3, and H4 proteins. Covalent modifications of these core histone proteins have been well-demonstrated to impact several aspects of nuclear function, such as transcription and DNA repair (Karlic et al., 2010). There are several known types of modifications that can occur, such as methylation, acetylation, phosphorylation, and ubiquitination; although not all have been found to be causal for neurological disease at this time. The modification of histones through methylation and acetylation, and the neurodevelopmental consequences of their aberrant activity in specific disorders, will be the focus of this review. While a number of genetic alterations affecting distinct encoded products have been implicated in different neurological conditions (Table 1), this review will focus on a select group of neurodevelopmental conditions linked to altered histone methylation or acetylation processes.

**Table 1:** Developmental Disorders Caused by Mutations in Histone Modifying Enzymes.

**Gene**	**OMIM #**	**Condition**	**Histone modification**
**Histone methyltransferases**			
KMT2A	605130	Wiedemann-Steiner syndrome	Mutant mice display normal global histone methylation; but decreased H4K5, 8, 12, 16 acetylation
KMT2D	147920	Kabuki syndrome 1	Mutant mice display decreased trimethylation of H3K4 in dentate granule cell layer
NSD1	117550	Sotos syndrome	Global reduction of H3K36 di-methylation in male germ cells
NSD2	194190	Wolf-Hirschhorn syndrome^*^	Reduced H3K36 methylation in heterozygous and homozygous KO mouse ESCs and embryonic zebrafish tissues
EHMT1	610253	Kleefstra syndrome^*^	In mouse hippocampus, cortex, cerebellum, and olfactory bulb increased H3K9 tri-methylation
EZH2	277590	Weaver syndrome	Mutant embryonic mouse tissues display decreased di- and tri-methylation of H3K27
**Histone Demethylases**			
KDM6A	300867	Kabuki syndrome 2	Mutant mouse neural crest cells display increased methylation of H3K27
KDM5C	300534	X-linked intellectual disability, Claes-Jensen type	No global changes in H3K4 methylation from *Kdm5c*-KO mouse neurons
PHF8	300263	X-linked intellectual disability, Siderius type	Not Determined
**Histone Acetyltransferases**			
CBP	180849	Rubinstein-Taybi syndrome (Type 1)	Hypoacetylated at H2A K5 (patient lymphoblastoid cells), H2B K5, K12, K15, K20 (mice and patient lymphoblastoid cells), H3 K14, K27 (CaMKIIα-Cre mice), and H4 K8 (CaMKIIα-Cre mice)
p300	613684	Rubinstein-Taybi syndrome (Type 2)	No observed alterations
KAT6A	616268	KAT6A syndrome	Hypoacetylated at H3K9
KAT6B	606170	Genitopatellar syndrome	Not determined
	603736	Say-Barber-Biesecker-Young-Simpson syndrome	Not determined
**Histone Deacetylases**			
HDAC4	600430	Brachydactyly mental retardation syndrome	No observed alterations

* *indicates disorders in which multiple genes have been implicated. References: (Nimura et al., 2009; Gervasini et al., 2013; Lopez-Atalaya et al., 2014; Iwase et al., 2016; Shpargel et al., 2017; Yu et al., 2017; Iacono et al., 2018; Lui et al., 2018; Shirane et al., 2020)*.

## Histone Methylation

Histone proteins can be methylated on arginine or lysine residues (Bannister et al., 2002; Bannister and Kouzarides, 2005). These modifications likely allow for the binding of specific regulatory proteins, which affect chromatin structure. This code is complex: Arginine residues may be mono- or di- methylated, while lysine residues may be mono-, di-, or tri-methylated (Bannister et al., 2002; Bannister and Kouzarides, 2005). This patterning allows for a vast array of different methylation state signatures (Bannister et al., 2002; Bannister and Kouzarides, 2005).

Arginine methylation typically occurs at amino acid residues 2, 8, 17, and 26 of H3, and residue 3 of H4 proteins (Klose and Zhang, 2007). Members of the family of arginine methyltransferase (PRMT1) enzymes carry out these modifications; their effects on chromatin structure can lead to either transcriptional activating or repressing consequences depending on context and code (Chen, 1999; Wang, 2001; Klose and Zhang, 2007). While histone methylation at arginine residues is recognized, neurodevelopmental conditions have not been identified to date that arise from systems involved in normal histone arginine modification. Rather, different neurodevelopmental conditions have been identified that stem from systems affecting histone lysine modifications.

**Figure 1:**
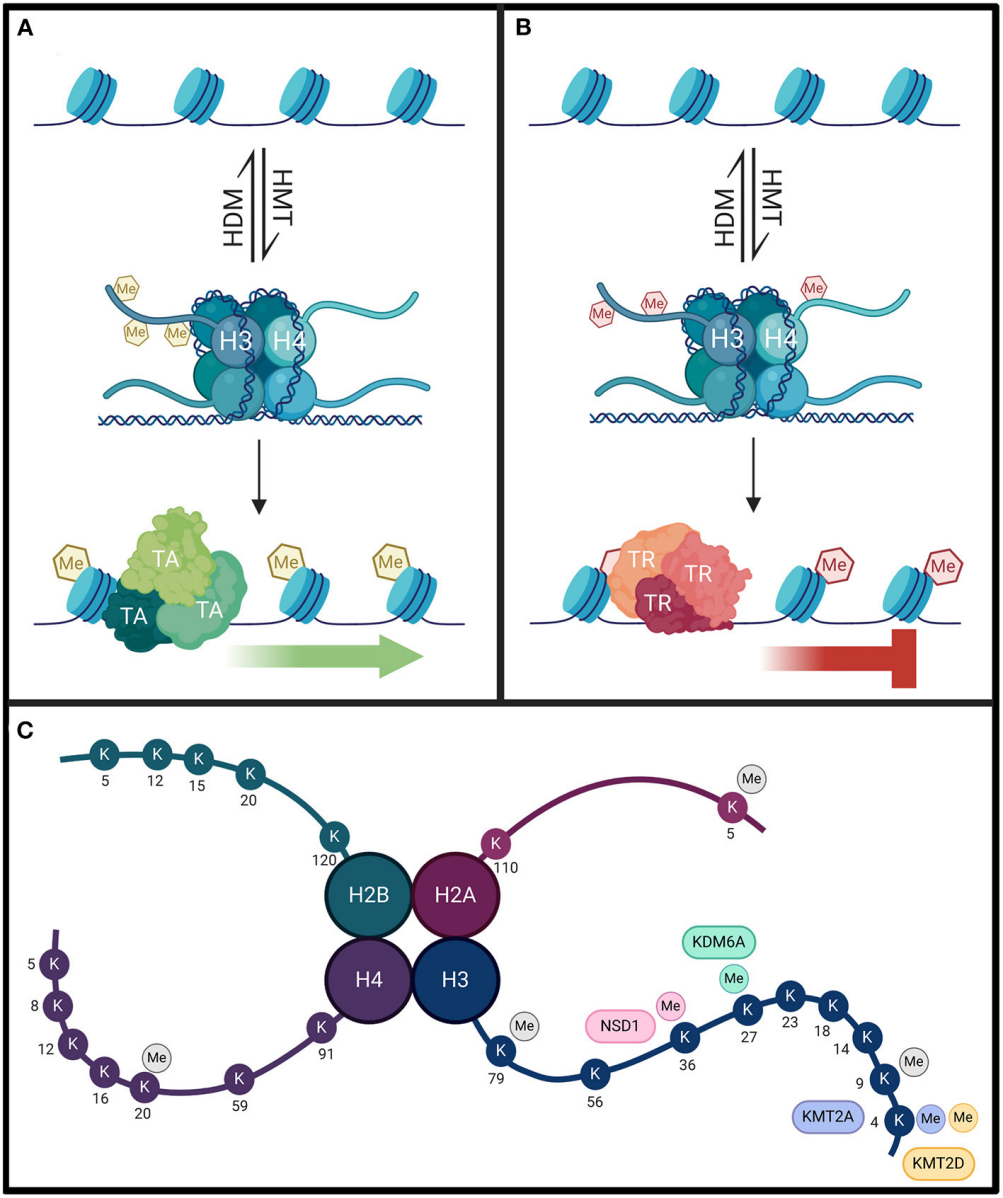
Histone methylation regulates gene expression through recruitment of different transcription factors rather than by directly altering chromatin structure. **(A)** When histone methyltransferases (HMT) methylate lysine or arginine residues on H3 and H4 associated with active chromatin (yellow hexagons), transcriptional activators (TA) can be recruited to those sites to promote gene expression. **(B)** However, when HMTs target different residues on H3 and H4 that are associated with chromatin repression, these methylated regions (red hexagons) can recruit transcriptional repressors to silence the gene. The transcriptional effects of histone methylation can be reversed by histone demethylases (HDM). Proper gene expression is dependent on the homeostasis of HMT and HDM enzyme activity. **(C)** Lysine methylation occurs primarily on H3 and H4. Each KMT/KDM will only target select residues, and the enzymes discussed have high specificity for these targets. Mutations that impair the function of these enzymes would be expected to affect the methylation status of these residues. Each enzyme and their lysine methylation sites are color coded. Other lysine methylation sites not specifically targeted by these enzymes are indicated in gray. Arginine methylation sites are not indicated in this figure.

Lysine methylation is carried out by the enzymes belonging to the disruptor of telomeric silencing 1-like (DOT1L) family, or to the family of SET domain containing proteins (Martin and Zhang, 2005; Klose and Zhang, 2007). Lysine methylation occurring at residues 4, 36, and 79 of histone H3 are generally associated with active chromatin, while methylation at residues 9 and 27 of histone H3, and/or lysine residue 20 of histone H4, are typically associated with inactive or repressed chromatin regions (Klose and Zhang, 2007). Methylation of histone proteins is not sufficient to alter their charge, and therefore, likely acts to recruit other effector proteins to specific chromatin sites rather than directly disrupting the contact between the histone complex and its associated DNA (Figure 1) (Klose and Zhang, 2007).

Histone lysine methylation is a dynamic process, and families of demethylase enzymes allow for the regulation of different histone methylation states (Black et al., 2012). Originally reported by Shi and colleagues, lysine specific demethylase 1 (LSD1) was the first histone demethylase described (Shi et al., 2003, 2004). LSD1 functions as a transcriptional corepressor by catalyzing oxidative demethylation of mono- and di-methylated lysine residues at positions 4 and 9 of H3 proteins (Shi et al., 2004; Metzger et al., 2005). LSD1 demethylase activity can also be influenced by other protein complexes, including the restin corepressor (CoREST) complex (Lee et al., 2005; Shi et al., 2005). Subsequently, the Jumonji C (JmjC)-domain-containing histone demethylase (JHDM) family of demethylase enzymes was identified, which contains the largest number of histone demethylases (Klose et al., 2006). These enzymes require a different set of cofactors [i.e., alpha-ketoglutarate, iron (Fe II)] (Tsukada et al., 2006). Unlike LSD1, these are able to modify all three histone methylation states (Klose et al., 2006). JHDMs have shown activity at a number of histone lysine residues, including H3K9 (Yamane et al., 2006) and H3K36 (Tsukada et al., 2006).

This dynamic process of methylating/demethylating histones can be disrupted by mutations in genes that encode the necessary catalytic proteins to produce these alterations. Mutations in histone methyltransferase and demethylase genes have been shown to be causal for several neurodevelopmental disorders, including, but not limited to, Wiedemann-Steiner, Kabuki, and Sotos syndromes (Kim et al., 2017).

### Conditions Associated With Impaired Histone Methyltransferase Function

#### Wiedemann-Steiner Syndrome

Wiedemann-Steiner syndrome (WDSTS; OMIM #605130) is a rare congenital malformation and neurodevelopmental disorder first described by Wiedemann et al. in 1989 and later by Steiner and Marques in 2000 (Wiedemann et al., 1989; Steiner and Marques, 2000). WDSTS is characterized by intellectual disability, language and motor delays, hypertrichosis cubiti, delayed bone age, and distinct craniofacial features (Koenig et al., 2010; Jones et al., 2012; Miyake et al., 2016; Li et al., 2018).

Through whole-exome-sequencing, Jones et al. determined that heterozygous mutations in the *KMT2A* gene (OMIM #159555) were causal for WDSTS in five patients (Jones et al., 2012). The *KMT2A* gene resides at chromosome 11q23 and encodes the KMT2A histone lysine methyltransferase (Jones et al., 2012). KMT2A is a SET domain-containing enzyme that catalyzes mono-, di-, and tri-methylation of H3K4. Known targets regulated by KMT2A include genes encoding several Hox and Wnt factors (Yu et al., 1995; Milne et al., 2002; Cosgrove and Patel, 2010; Jones et al., 2012). Methylated H3K4 is typically found at enhancers and promoters of genes being actively transcribed, with both methylation status (mono-, di-, or tri-) and density correlating with the level of transcriptional activity (Heintzman et al., 2007, 2009; Kim et al., 2017).

Several mutations of *KMT2A* have been reported, which include single nucleotide missense mutations, non-sense mutations, insertion/deletion alterations that cause frame-shift mutations, and mutations affecting splice site sequences (Li et al., 2018). Most of the known mutations alter the reading frame and introduce termination codons in a non-terminal exon. Such inappropriate termination codons are likely to activate non-sense-mediated mRNA decay pathways (Jones et al., 2012), and if indeed non-sense mediated decay does target these non-sense mutation transcripts, the resulting condition would likely arise due to haploinsufficiency (Yu et al., 1995; Jones et al., 2012; Li et al., 2018). The mechanism associated with missense mutations of *KMT2A* remain unclear, however, and both loss of function and dominant negative mechanisms have been hypothesized to cause WDSTS (Stellacci et al., 2016; Lebrun et al., 2018; Li et al., 2018).

A mouse model harboring a lacZ insertion into exon 3 of *Kmt2a* has been generated for preclinical assessments of WDSTS (Yu et al., 1995; Kim et al., 2007; Gupta et al., 2010). *Kmt2a* null mutations are embryonic lethal in mice (Yu et al., 1995), although heterozygous mice are viable and display phenotypes that phenocopy clinically-relevant issues often seen in patients. *Kmt2a* heterozygous mice were found to have skeletal malformations, reductions in growth, and haematopoietic abnormalities, including anemia and thrombocytopenia (Yu et al., 1995). These mice also showed substantial deficits in long-term contextual fear learning (Gupta et al., 2010). Targeted ablation of *Kmt2a* in the postnatal forebrain and adult prefrontal cortex (floxed exon 3 and 4) has been reported to produce profound cognitive deficits, increase anxiety-like behaviors, deficits in working memory, and impaired synaptic plasticity (Jakovcevski et al., 2015). Perhaps surprisingly, neither global histone methylation nor H3K4 methylation were found to be altered in brain of *Kmt2a* heterozygous mice at either postnatal day 0 or in adulthood (Kim et al., 2007). However, these mice do display decreases in histone acetylation at H4K5, 8, 12, and 16 (Kim et al., 2007). As Kmt2a normally interacts with HDAC1/2 complexes (van der Vlag and Otte, 1999; Xia et al., 2003), the altered H4K5 acetylation could stem from the lack of proper Kmt2a-directed HDAC1/2 activity. In addition, the expression of the homeodomain transcription factor Meis2 is reduced in *Kmt2a*-ablated neurons. This alteration may be linked to pathogenesis, as the selective knockdown of *Meis2* in the prefrontal cortex of mice using RNA interference produced similar deficits in working memory as those seen in *Kmt2a* mutants (Jakovcevski et al., 2015). While the precise role of Meis2 in WDSTS pathogenesis remains unclear, restoring Meis2 function has been proposed as a potential strategy for translational investigation of WDSTS (Kim et al., 2017).

#### Kabuki Syndrome

Kabuki syndrome (KS; OMIM #147920) is an intellectual disability and multiple congenital malformation disorder first described by Niikawa et al. (1981) and Kuroki et al. (1981). An international consensus panel has outlined the diagnostic criteria for KS to include an individual with infantile hypotonia, developmental delay or intellectual disability, and one or both of the following: (1) a pathogenic, or likely pathogenic mutation of *KMT2D* or *KDM6*A; (2) dysmorphic features associated with KS (for full details see Adam et al., 2019) (Adam et al., 2019). KS has an estimated incidence rate of ~1 in 32,000 in Japan (Niikawa et al., 1988).

Heterozygous mutations in *KMT2D* (OMIM #602113) have been identified as causal for KS through whole-exome and sanger sequencing (Ng et al., 2010). KMT2D is a SET domain containing histone lysine methyltransferase first cloned in 1997, whose encoding gene resides on chromosome 12q13.12 (Prasad et al., 1997). KMT2D is a H3K4 tri-methyltransferase, whose activities are typically associated with enhancing local gene expression and are critical for proper cell differentiation (Lee et al., 2013; Van Laarhoven et al., 2015). A vast number of KS-associated mutations in *KMT2D* have been identified; the majority being non-sense and frameshift mutations (Ng et al., 2010; Hannibal et al., 2011; Li et al., 2011; Miyake et al., 2013a; Micale et al., 2014). These mutations were predicted to produce truncated proteins that do not include the SET domain necessary for the protein's methylation abilities (Ng et al., 2010; Hannibal et al., 2011; Miyake et al., 2013a), or may activate non-sense-mediated mRNA decay pathways (as discussed previously). Either mechanism would support the hypothesis of KMT2D haploinsufficiency as a causal mechanism for KS (Hannibal et al., 2011).

Knockdown of *Kmt2d* in zebrafish using morpholino antisense oligonucleotides resulted in substantial craniofacial defects and viscerocranial hypoplasia (Van Laarhoven et al., 2015). The effects of this knockdown on neurodevelopment were assessed in the embryonic stage, and brain cross-sections showed global volume reductions compared to wildtype. Cell layer thickness was found to be reduced in the optic tectum, midbrain tegmentum, hypothalamus, and, to a lesser extent, the medulla oblongata and hindbrain (Van Laarhoven et al., 2015). The presence of morphological abnormalities, such as elongated nuclei, in cells found in the central forebrain and midbrain regions has also been reported. These areas were also shown to contain much larger populations of cells expressing sox2 (a marker of neural precursor cells) and very diminished number of cells expressing the neuronal post-mitotic marker huc. Together, these observations suggest the differentiation of neural precursor cells is inhibited by the absence of Kmt2d (Van Laarhoven et al., 2015). In mice, the complete knockout of *Kmt2d* was found to be embryonically lethal (Lee et al., 2013). However, a mouse model has been generated in which the SET domain of *Kmt2d* has been substituted in frame with a beta-Geo cassette including its own termination codon, 3' untranslated region, and poly-adenylation signal. This mouse expresses a truncated protein that lacks methyltransferase activity but should retain its more proximal amino terminal domains (Bjornsson et al., 2014). Mice homozygous for this mutation become non-viable during embryonic development by ED12. Mice heterozygous for this mutation show a reduction in neurogenesis of the granule cell layer of the dentate gyrus and a decrease of hippocampal dentate gyrus volume. Consistent with this observation, these mice display deficits in the Morris water maze, contextual fear learning, and novel object recognition tests, suggesting impairments in hippocampal memory (Bjornsson et al., 2014). At the cellular level, these phenotypic alterations correlated with significant decreases in H3K4 trimethylation levels in the hippocampal dentate granule cells of these mice (Bjornsson et al., 2014). To date, H3K4 methylation status in patient-derived material or cells has yet to be investigated.

A less frequent cause of KS, seen in <5% of patients (Banka et al., 2015), stems from mutations in the *KDM6A* (also known as *UTX*) gene (OMIM #300128) (Lederer et al., 2012; Van Laarhoven et al., 2015). The *KDM6A* gene resides at chromosome Xp11.3 and encodes a histone-demethylase that targets mono-, di-, and tri- methylated H3K27 (Hong et al., 2007; Lan et al., 2007; Lederer et al., 2012). Interestingly, *Kdm6a* has been shown to escape X-inactivation (Greenfield et al., 1998), and display some sex dependent differences in magnitude of expression in mice (Xu et al., 2008). The activity of Kdm6a is primarily associated with gene silencing (Hübner and Spector, 2010; Margueron and Reinberg, 2011). Its regulation of methylation state has been shown to influence gene expression and developmental processes, such as transitions in cell lineage (Miller et al., 2010; Wang et al., 2010), and is critical for neural tube development in mice (Shpargel et al., 2012). Several *KDM6A* mutations have been identified in KS patients, which include deletions, frameshifts, mutations affecting splice site junctions, and non-sense mutations (Lederer et al., 2012, 2014; Miyake et al., 2013b; Banka et al., 2015; Van Laarhoven et al., 2015). Each of these mutations would likely generate a non-functional product or promote non-sense-mediated decay, suggesting pathogenesis likely stems from KDM6A haploinsufficiency (Lindgren et al., 2013).

Similar to what was observed in *Kmt2d* models, morpholino knockdown of *Kdm6a* resulted in significant craniofacial abnormalities in zebrafish (Lindgren et al., 2013; Van Laarhoven et al., 2015), as well as reductions in global brain volume and cell layer thickness of the optic tectum, hypothalamus, midbrain tegmentum, and medulla oblongata (Van Laarhoven et al., 2015). The co-administration of wild-type *hKDM6A* mRNA was found to partially reverse these cell layer reductions in the morpholino knockdown mutants (Van Laarhoven et al., 2015). In normal embryonic development in mice, the expression of *Kdm6a* is high in the ventricular zone of the caudal neural tube, the anterior region of the neural tube, in neural crest cells, and in somites at E8.5, and in the cortex at E11.5 (Lee et al., 2012). Knockout of *Kdm6a* in embryonic female mice is lethal at mid-gestational periods, with severe defects in early developmental patterning being observed (including failure of neural tube closure). However, male embryos expressing the same mutation develop to term, but are underweight at birth with only 25% surviving into adulthood. Females heterozygous for this mutation were found to be viable and fertile (Lee et al., 2012; Shpargel et al., 2012). Consistent with expectations that would stem from impaired Kdm6a function, increased methylation at H3K27 has been reported in neural crest cells isolated from these mutant mice (Shpargel et al., 2017).

#### Sotos Syndrome

Sotos syndrome (OMIM #117550) is an autosomal dominant, neurologic disorder first described by Sotos et al. (1964). Sotos syndrome is characterized by excessive pre- and post-natal growth, advanced bone age, distinct facial features, macrocephaly, neurodevelopmental and intellectual delay, and in some instances seizures (Sotos et al., 1964; Kurotaki et al., 2002). In light of the discovery of causal mutations associated with Sotos syndrome, the Childhood Overgrowth Collaboration Consortium found an overgrowth in occipitofrontal head circumference, facial dysmorphism, and learning disability were defining characteristics, and an array of associated features, such as advanced bone age, macrocephaly, hypotonia, seizures, scoliosis, cardiac defects, and neonatal jaundice (Tatton-Brown et al., 2005). Neuroimaging studies have identified common ventricular abnormalities, including prominence of the trigone and occipital horns, and ventriculomegaly. Other imaging findings include enlarged supratentorial and posterior fossa extracerebral fluid space, anomalies of the corpus callosum and midline structures, and in a small percentage of cases gray matter heterotopias (Schaefer et al., 1997). The prevalence of Sotos syndrome is currently unclear and under investigation. Treatment of Sotos syndrome currently involves symptomatic management (Baujat and Cormier-Daire, 2007).

Mutations in the nuclear receptor-binding SET domain protein 1 (*NSD1*; OMIM #606681) were found to be causal for Sotos syndrome (Kurotaki et al., 2002) and mutations of *NSD1* may account for more than 75% of the reported cases (Baujat and Cormier-Daire, 2007). Different types of *NSD1* mutations have been reported, which include non-allelic homologous recombination, large and small scale deletions, frameshifts, as well as missense and non-sense mutations (Kurotaki et al., 2002; Douglas et al., 2003; Türkmen et al., 2003; van Haelst et al., 2005; Visser et al., 2005; Kaminsky et al., 2011). The *NSD1* gene is located at chromosome 5q35 (Jaju et al., 2001) and encodes a SET domain containing histone lysine methyltransferase (Huang et al., 1998). NSD1 is a highly specific mono- or di-methyltransferase of H3K36 (Li et al., 2009; Lucio-Eterovic et al., 2010), and has been shown to associate with the promoter regions of a number of genes across the genome to regulate their regional methylation signatures (Lucio-Eterovic et al., 2010). The majority of mutations identified are predicted to disrupt the reading frame in a way that causes early translational termination and/or activates non-sense-mediated decay (Tatton-Brown et al., 2005), suggesting haploinsufficiency is the likely cause of pathogenesis (Kurotaki et al., 2002). Mutations known to be causal for Sotos syndrome that localized to the plant homeodomain (PHD) regions of the encoded protein diminished NSD1 binding to specific methylated sites (H3K4 and H3K9), and abrogated cofactor recruitment (e.g., Nizp1), which collectively lead to impairments in normal transcriptional regulation (Pasillas et al., 2011). NSD1 has also been found to interact with RNA polymerase II, possibly through NSD1-methylation dependent recruitment to promoter regions. The loss of NSD1 also leads to reductions in methylated H3K36 and gene expression *in vitro* (Lucio-Eterovic et al., 2010).

The *NSD1* gene is highly conserved between human and mouse (83% amino acid identity) (Kurotaki et al., 2001) and mouse models have been developed for targeted knockdown using Cre-loxP recombination (Rayasam et al., 2003). Complete *Nsd1* knockout in mice is embryonic lethal (by E10.5), with the *Nsd1* knockout embryos displaying significant alterations in endoderm, mesoderm, and neurectoderm pattern formation at E8.0, and a significant increase in terminal deoxynuleotidyltransferase-mediated dUTP-biotin nick-end labeling (TUNEL)-positive cell labeling to suggest increased levels of apoptosis (Rayasam et al., 2003). Unlike the consequences of heterozygous *NSD1* mutations in patients, however, mice heterozygous for a *Nsd1* null allele are viable and fertile, and display normal growth rates (Rayasam et al., 2003). A second mouse model has also been generated that houses a microdeletion that ablated 36 genes including *Nsd1* on mouse chromosome 13 (which is syntenic with the *NSD1* region on human chromosome 5q35.2 – q35.3). Mice heterozygous for this mutation displayed growth reductions and impairments in long-term memory retention (Migdalska et al., 2012), although the specific role played by Nsd1 in these phenotypic consequences remains unclear. Germ cells isolated from male Nsd1-deficient mice display a global reduction in di-methylation of H3K36 (Shirane et al., 2020).

Although less common, mutations in the adenomatous polyposis coli 2 (*APC2*) gene (OMIM #612034) – a gene expressed in post-mitotic neurons that regulates cytoskeletal structure, axon guidance and neuronal migration – can also cause a Sotos syndrome variant condition (Almuriekhi et al., 2015). The *APC2* gene is normally targeted by NSD1 regulation, and knockdown of Nsd1 using shRNA significantly reduced the expression of *Apc2* mRNA and protein in primary mouse cortical cells (Almuriekhi et al., 2015), suggesting there is a direct link between Nsd1 and Apc2 cooperative function. Consistently, transfection of Apc2-miRNA into cortical progenitor cells (E15.5) resulted in abnormal migration, with a large proportion of neurons remaining in the lower cortical layers (Almuriekhi et al., 2015). This was highly similar to the pattern seen in the same system after treatment with *Nsd1*-miRNA (Almuriekhi et al., 2015), and in the *Apc2* null mouse brain (Shintani et al., 2012). Importantly, these deficits arising from *Nsd1-*miRNA expression were rescued by the co-administration of an *Apc2*-expression plasmid (Almuriekhi et al., 2015). This further indicates the critical role played by Nsd1 in neuronal migration, and proper neurodevelopment requires its regulation of *Apc2* expression. Mutations affecting either factor disrupt this normal signaling cascade and result in Sotos syndrome.

## Histone Acetylation

Histone acetylation is another critical epigenetic process that defines chromatin structure. Histone acetylation regulates numerous cell processes including cell-cycle progression, differentiation, and metabolism, largely by its role in orchestrating transcriptional responsiveness (Podobinska et al., 2017). Histone acetylation is facilitated by histone acetyltransferases (HATs), and conversely these modifications are removed by histone deacetylases (HDACs) (Gräff and Tsai, 2013). Alternative nomenclature for these enzymes can include lysine acetyltransferases and deacetylases (KAT and KDAC, respectively), as their enzymatic activities are not limited to histone proteins (Seto and Yoshida, 2014).

HATs can be classified into three main groups based largely on homology. The first group is the Gcn5-related N-acetyltransferase (GNAT) family, which is arguably the best understood and includes Gcn5 – the first HAT discovered – its close relatives (ex. PCAF), and the related HATs HAT1, ELP3, and HPA2 (Sterner and Berger, 2000; Bonnaud et al., 2016). The second group of HATs is referred to as CBP/p300, and consists, as the name suggests, of the ubiquitously expressed CREB-binding protein (CBP) and its close relative p300 (Sterner and Berger, 2000; Bonnaud et al., 2016). The third group of HATs is the MYST family, which is an acronym of its founding members MOZ, Ybf2/Sas3, Sas2, and Tip60 enzymes (Sterner and Berger, 2000; Bonnaud et al., 2016). In addition to targeting histones, HATs can act as scaffolds for promoter-binding transcription factors, and acetylate these factors to alter their activity (Sterner and Berger, 2000). Additional proteins with HAT activity also exist, and include nuclear receptor co-activators, such as SLC-1, NCoA-3, and CLOCK (Sterner and Berger, 2000; Bonnaud et al., 2016). Lastly, certain transcription factors can also possess HAT activities, with TFIID and TFIIIC being known examples (Sterner and Berger, 2000; Bonnaud et al., 2016). HATs typically act either as nuclear modifiers of nucleosomes (A-type) or cytoplasmic modifiers of newly synthesized histones (B-type) (Sterner and Berger, 2000; Bannister and Kouzarides, 2011).

HDACs can be subdivided into four classes. Class I HDACs consists of HDACs 1–3, and 8, whose functions are restricted exclusively to the nucleus (Kouzarides, 2007; Bonnaud et al., 2016). Class IIa HDACs (4, 5, 7, and 9) are shuttled between the nucleus and cytoplasm, but possess no intrinsic deacetylase activity and are thought to act as scaffolds for other co-repressor systems (Mielcarek et al., 2015; Bonnaud et al., 2016). Class IIb enzymes (6 and 10) largely function outside the nucleus, where they mediate deacetylation of cytosolic proteins (Seto and Yoshida, 2014; Bonnaud et al., 2016). Class III HDACs consists of the sirtuin (SIRT) subfamily of enzymes that reside within the nucleus, cytosol, and mitochondria (Kouzarides, 2007; Bonnaud et al., 2016). Class IV HDACs presently consists of only the nuclear HDAC11 (Seto and Yoshida, 2014; Bonnaud et al., 2016). Deacetylation reactions are zinc dependent for Class I, II, and IV enzymes, while Class III enzymes are dependent on NAD^+^ as a cofactor (Seto and Yoshida, 2014; Bonnaud et al., 2016).

**Figure 2:**
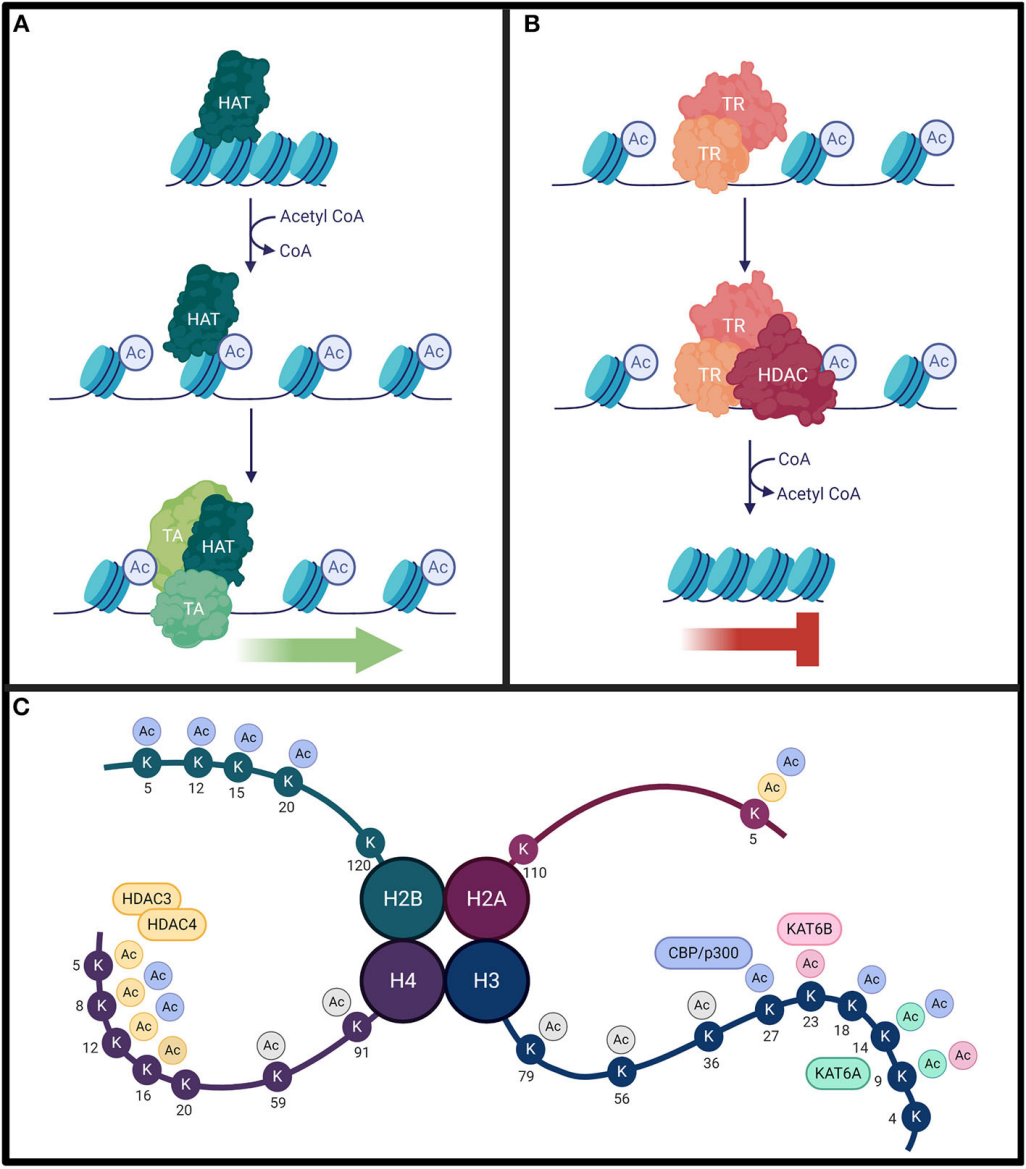
**(A)** Histone acetyltransferases (HAT) will acetylate histone lysine residues using acetyl CoA cofactor (blue circles). This weakens the electrostatic interactions between positively-charged histones and negatively-charged DNA to loosen chromatin structure. This results in DNA exposure, allowing for the recruitment of transcriptional activators (TA). **(B)** Conversely, transcriptional repressor (TR) complexes can interact with histone deacetylases (HDAC) to remove these modifications. This strengthens the electrostatic interactions between the DNA and the histones, resulting in compact chromatin, inhibiting transcription. The homeostasis between histone acetylation and deacetylation is critical for proper gene expression. **(C)** Lysine acetylation can occur on all four histone subunits. Each HAT/HDAC has preferred target sites. While KAT6A and KAT6B acetylate residues with high specificity, CBP and HDAC4/HDAC3 complexes have a broader range of targets. The lysine residues targeted by each enzyme are color coded. There is some target redundancy between HAT/HDACs, and other histone (de)acetylating enzymes can also target common sites (not indicated on figure). Additional lysine acetylation sites not specifically targeted by these enzymes are indicated in gray.

Histone acetylation patterns influence transcription by altering chromatin structure. Mechanistically, lysine residues residing toward the amino terminus of histone proteins are positively charged at physiological pH. These charges form an electrostatic interaction with the negative charge of DNA, which tightens the association of the DNA with the histone protein and encourages chromatin compaction. When these lysine sites are targeted for acetylation, the result is the neutralization of the positive charge, a weakening of the electrostatic interactions between histones and DNA, and a relaxation of chromatin (Figure 2) (Gräff and Tsai, 2013; Podobinska et al., 2017). Deacetylation, conversely, exposes the positive charges, promotes stronger electrostatic interactions, and favors condensed chromatin (Gräff and Tsai, 2013; Podobinska et al., 2017). Histone acetylation promotes transcription factor recruitment to the exposed DNA, whereas deacetylation restricts access (Bannister and Kouzarides, 2011). While HATs primarily modify histones H3 and H4 on the exposed N-terminal tail, acetylation can occur at core and tail regions of all histones (Podobinska et al., 2017). The hallmark lysine residues acetylated on H3 are K9, K14, K18, K23, and K56, while on H4 the hallmark residues are K5, K8, K12, and K16 (Podobinska et al., 2017; Sheikh and Akhtar, 2019). In addition, the pattern of acetylation established can provide a recognition site for certain transcription factors that also facilitate transcription (Kouzarides, 2007). Ultimately, the balance of HAT and HDAC activity throughout genomic segments determines transcriptional accessibility and activity of many dynamically expressed genes (Kouzarides, 2007).

Much like histone methylation, when an imbalance in these systems occurs it can have detrimental effects on neurodevelopment. This can arise from mutations in key acetylating or deacetylating enzymes. Several gene mutations have been identified that affect these systems and are causal for neurodevelopmental diseases. Examples include Rubinstein-Taybi Syndrome, KAT6A syndrome, Genitopatellar syndrome, Say-Barber-Biesecker-Young-Simpson syndrome and Brachydactyly mental retardation syndrome.

### Conditions Associated With Impaired Histone Acetyltransferase Function

#### Rubinstein-Taybi Syndrome

Rubinstein-Taybi syndrome (RTS; OMIM #180849) is a neurodevelopmental disorder affecting 1 in ~125,000 live births (Hutchinson and Sullivan, 2015). RTS is caused by mutations in the KAT3 subfamily member *CREBBP*, which encodes CREB binding protein (CBP) (Type 1 RTS) or in subfamily member *EP300*, which encodes p300 (Type 2 RTS) (Korzus, 2017; López et al., 2018). Approximately 55% of RTS cases stem from *CREBBP* mutations, while ~10% of RTS patients result from *EP300* mutations (Korzus, 2017). Both of these mutations cause deficiencies in histone acetylation activity/efficiency. The autosomal dominant mutations of either gene largely occur *de novo* in germline cells, and the cellular consequences arise either through enzymatic haploinsufficiency or via a dominant negative mechanism (Barco, 2007; Park et al., 2014). The genetic cause of the remaining third of RTS cases is currently unknown (López et al., 2018). The diagnosis of RTS is based predominantly on clinical presentation, often within the 1st year of life (Korzus, 2017). Features of RTS include broad thumbs and halluces, severe cognitive impairment, facial abnormalities, and psychomotor delay (Park et al., 2014; Hutchinson and Sullivan, 2015). Some patients may also present with seizures, spinal deformities, syndactyly, and congenital heart abnormalities (Lopez-Atalaya et al., 2014; Hutchinson and Sullivan, 2015). RTS patients have also been found to be more susceptible to the development of CNS cancers later in life (Park et al., 2014). Cognitive impairments occur in 99% of patients harboring *CREBBP* mutations and 94% of those with *EP300* mutations, with IQ scores ranging from <25 to 80 (Korzus, 2017; López et al., 2018). The remaining phenotypes discussed above tend to be observed more frequently and with greater severity in patients with *CREBBP* mutations as compared to those with *EP300* mutations (Korzus, 2017).

*CREBBP* is located on chromosome 16p13.3 and mutations consist of either chromosomal rearrangements within this region or mutations within the *CREBBP* gene itself (Petrij et al., 1995; Barco, 2007). Translocations, breakpoints, insertions, and microdeletions of chromosome 16 in this region can all contribute to RTS, and larger rearrangements generally associate with an increased severity of the condition (Petrij et al., 1995; Lopez-Atalaya et al., 2014). Various mutations within the *CREBBP* gene have been reported including missense, non-sense, frameshift, insertion, deletions, and mutations located near the splice-site junctions (Lopez-Atalaya et al., 2014; Korzus, 2017). Deletion mutations can occur along the entire length of the gene or affect specific parts, although mutations in reading frame exons, that encode the histone acetyltransferase domain, are most common (Barco, 2007; Korzus, 2017). The same is true for *EP300*, which is located on chromosome 22q13.2; both with regards to chromosome aberrations and coding sequence mutations (Korzus, 2017). CBP and p300 target numerous lysine residues on all four histones: H2A (K5), H2B (K5, K12, K15, K20), H3 (K14, K18, K27), and H4 (K8, K12) (Bedford and Brindle, 2012; Valor et al., 2013; Lipinski et al., 2020). CBP and p300 are critical in many nuclear processes, due both to their respective HAT activities, and their ability to interact with over 400 transcription factors that effectively compete for the proportionally limited amount of CBP/p300 present in the cell (Dyson and Wright, 2016). While there is some overlap between the target genes of CBP and p300, these two HATS also display some distinct targets and functions. Thus, mutations in either protein cannot be completely accommodated by the other (Lopez-Atalaya et al., 2014; Korzus, 2017).

Various mouse models of RTS have been developed which target both *Crebbp* and *Ep300*. The first models created were knockout mice in which specific domains of either gene were targeted for ablation (e.g., their respective KAT, KIX, and CH1 domains) (Barco, 2007; Lopez-Atalaya et al., 2014). For both *Crebbp* and *Ep300*, homozygous mutants expressing these disruptions were embryonic lethal (Barco, 2007; Lopez-Atalaya et al., 2014). Interestingly, mice jointly heterozygous for both *Crebbp* and *Ep300* were also not viable (Lopez-Atalaya et al., 2014). The *Crebbp* or *Ep300* heterozygous mice mirrored many phenotypes observed in patients, including inhibited growth, select skeletal deformities, and memory impairments (Barco, 2007), although the characteristic broad halluces seen in RTS patients are not evident in these mice (Lopez-Atalaya et al., 2014). Examination of histone acetylation status in *Crebbp* heterozygous mice revealed global hypoacetylation of H2B, whereas the other histone subunits were unaffected (Alarcón et al., 2004). In contrast, *EP300* heterozygous mice showed no change in acetylation status (Oliveira et al., 2007; Viosca et al., 2010). Knock-in mice have also been generated that express specific point mutations for both *Crebbp* and *Ep300*, permitting examination of the role specific domains of each protein have on overall function (Lopez-Atalaya et al., 2014). Conditional knockouts have also been generated using Cre-loxP systems that target specific regions of the hippocampal formations, such as the dentate gyrus and CA1 using the CamKIIα promoter (Barco, 2007; Barrett et al., 2011; Lopez-Atalaya et al., 2014). CaMKIIα-Cre/*Crebbp* mice display impaired short- and long-term memory, and acetylation deficiencies at H2BK12, H3K14, H3K27, and H4K8 (Barrett et al., 2011; Lipinski et al., 2020). A tetracycline-induced transgenic mouse was developed to allow for temporal control of *Crebbp* expression (Barco, 2007; Lopez-Atalaya et al., 2014). In these mice, the HAT domain was inactive, but Cbp could still interact with its partner transcription factors (Barco, 2007; Lopez-Atalaya et al., 2014). These mice also presented with long-term memory impairments (Barco, 2007). These region-specific ablations resulted in learning deficits and diminished LTP, which mirror what is seen in RTS patients (Gräff and Tsai, 2013; Lopez-Atalaya et al., 2014). Overall, these mouse models indicate that H2B acetylation is the site most predominantly affected by CBP deficiency, but that intact p300 cannot fully compensate for the CBP deficiency at H2B. This diminished H2B acetylation pattern was also found in patient-derived lymphoblastoid cultures expressing *CREBBP* mutations (Lopez-Atalaya et al., 2012), although these cells also displayed hypoacetylated H2A (but not H3 and H4). Intriguingly, this hypoacetylation in H2A and H2B was rescued by treating the cells with an HDAC inhibitor (Lopez-Atalaya et al., 2012), suggesting a possible avenue for translational development.

#### KAT6A Syndrome

KAT6A syndrome (OMIM # 616268) is a rare autosomal dominant disorder first described in 2015 by Arboleda et al. and Tham et al. (Arboleda et al., 2015; Tham et al., 2015). Intellectual disability and global developmental delay are observed in patients, concomitant with notable speech delays (Arboleda et al., 2015; Tham et al., 2015). This oromotor dyspraxia is universal in KAT6A syndrome, with more marked impairments in expressive language than receptive language (Kennedy et al., 2019). Clinical features are variable between patients, however, hypotonia, microcephaly, craniofacial dysmorphisms, congenital cardiac defects, gastrointestinal problems, feeding difficulties, strabismus, and sleep disturbances have been reported (Arboleda et al., 2015; Millan et al., 2016; Kennedy et al., 2019). Delayed myelination has also been observed in some patients, although was found to resolve over time (Millan et al., 2016; Alkhateeb and Alazaizeh, 2019).

KAT6A syndrome arises from mutations in the gene encoding the lysine acetyltransferase *KAT6A* (also known as *MYST3, MOZ*), which is located on chromosome 8p11.21 (Tham et al., 2015). The *KAT6A* gene locus consists of 18 exons, which encodes for a protein of 2004 amino acids that houses five domains: H15 nuclear localization, PHD, KAT, acidic Glu/Asp-rich, and Ser/Met-rich transactivation domain (Klein et al., 2014; Tham et al., 2015). Key residues in the KAT domain have been identified as E680 and C646, wherein the former actively interacts with the acetyl CoA cofactor (Klein et al., 2014). KAT6A primarily targets H3K9, and to a lesser extent H3K14, to loosen chromatin structure as described above (Yang, 2015). In addition, KAT6A can complex with factors, such as BRPF1/2/3, hEAF6, and ING5, or interact with RUNX1/2 to activate the transcription of a number of genes, such as Hox (Voss et al., 2009; Yang, 2015). Truncating, frameshift, and non-sense mutations are the most common mutations causal for KAT6A syndrome, although missense mutations, and mutations occurring at splice junction sites have been identified in some patients (Tham et al., 2015; Millan et al., 2016; Kennedy et al., 2019). Mutations in the KAT domain itself are infrequent; mutations are rather predominantly found in the more distal exons 16 to 18, that encode for the acidic Glu/Asp-rich domain and the Ser/Met-rich domain (Tham et al., 2015; Kennedy et al., 2019). Specific non-sense mutation hotspots at positions 1,019, 1,024, and 1,129 have been identified that account for about 20% of the total KAT6A syndrome cases (Tham et al., 2015; Kennedy et al., 2019). Interestingly, patients with distal exon mutations exhibit more severe symptoms than those with mutations in more proximal exons 1–15 (Kennedy et al., 2019). This is postulated to be a result of KAT6A transcript fate. If the mutation is proximal, the transcript undergoes nonsense-mediated decay, and symptoms arise through haploinsufficiency (Kennedy et al., 2019). It has been hypothesized that transcripts with mutations in exons 16, 17, and terminal exon 18, however, may not be degraded, and consequently encode a truncated protein that acts in a dominant-negative or gain-of-function manner (Kennedy et al., 2019). At present, however, experiments to test this hypothesis remain to be conducted.

Various animal models have been generated for investigating the role of Kat6a in development. Initially, Kat6a deficiency was examined in mutant zebrafish obtained through ENU mutagenesis, which resulted in aberrant craniofacial development (Miller et al., 2004; Crump, 2006). As mentioned above, Kat6a is a key player in the proper expression of the *Hox* genes that define segmental identity (Crump, 2006; Vanyai et al., 2019). In these Kat6a-deficient zebrafish, the second pharyngeal arch of the cranial neural crest was replaced by a duplicated jaw structure (Miller et al., 2004; Crump, 2006). Loss of Kat6a function in zebrafish appears to exclusively affect the head, whereas rodents are more globally affected (Tham et al., 2015). Two *Kat6a* targeted mouse models have also been generated (Voss et al., 2012). To model early exon deletions in *Kat6a*, exons 3-7 were flanked with loxP sites and removed globally via Cre recombinase (Voss et al., 2009). In contrast to findings in humans, early exon mutations were more severe than C-terminal mutations in mice, with complete null mice displaying embryonic lethality between E14.5 and birth (Voss et al., 2012). These homozygous early exon *Kat6a* knockout mice aged E10.5 days had elongated necks with an additional eighth cervical vertebra, and one fewer thoracic vertebra than heterozygotes and wild-types (Voss et al., 2009). This was attributed to *Hox* gene repression, as the histones of those loci were hypoacetylated instead of being tri-methylated at H3K9 (Voss et al., 2009). Consistently, this same histone modification pattern was observed in fibroblasts from KAT6A syndrome patients (Arboleda et al., 2015). The other model was generated by an in-frame insertion of a neomycin phosphotransferase sequence in exon 16, near the carboxyl terminus (Thomas et al., 2006). These mice survived throughout gestation, but remained severely affected and died within hours after birth (Voss et al., 2012). Both mouse models presented with cardiac defects, caused by *Tbx1* repression in the absence of Kat6a (Voss et al., 2012). In addition, both early and late exon *Kat6a* knockouts exhibited a loss of hematopoietic stem cells, and craniofacial dysmorphisms, including cleft palates (Voss et al., 2012; Vanyai et al., 2019). Except for mild craniofacial dysmorphisms and heart defects, these mouse models do not appear to phenocopy the human condition.

#### Genitopatellar Syndrome and Say-Barber-Biesecker-Young-Simpson Syndrome

Genitopatellar syndrome (GPS; OMIM #606170), first reported in 1988 (Goldblatt et al., 1988), is a condition characterized by neurological, skeletal, and genital abnormalities. Say-Barber-Biesecker-Young-Simpson syndrome (SBBYSS; OMIM#603736) was first described in 1986 (Ohdo et al., 1986; Campeau et al., 2012) and shares many overlapping clinical features with GPS, although somewhat less severe (Campeau et al., 2012). Both of these autosomal dominant conditions result from mutations in the *KAT6B* gene (previously named *MORF* and *MYST4*) that resides on chromosome 10q22.2 (Lonardo et al., 2019). However, the mutations in GPS and SBBYSS occur at different loci on the *KAT6B* gene (Lonardo et al., 2019). Intellectual disability, developmental delay, congenital heart defects, thyroid and dental anomalies, hypotonia, feeding difficulties, and hearing loss are present in both GPS and SBBYSS (Campeau et al., 2012; Vlckova et al., 2015; Lonardo et al., 2019). Genital and anal anomalies are common for both sexes in GPS, whereas they are rare, mild, and restricted to males in SBBYSS (Campeau et al., 2012; Lonardo et al., 2019). Patients with GPS also exhibit patellar hypoplasia or agenesis, flexion contractures of the hips and knees, club-foot, hydronephrosis, renal cysts, agenesis of the corpus callosum, and microcephaly (Campeau et al., 2012; Vlckova et al., 2015; Lonardo et al., 2019) While both conditions present with cleft palates, prominent cheeks and bulbous noses, the craniofacial dysmorphisms of SBBYSS also include mask-like, immobile facies, blepharophimosis, and ptosis (Vlckova et al., 2015; Lonardo et al., 2019). In addition, SBBYSS patients typically have long fingers and toes (Campeau et al., 2012; Vlckova et al., 2015). Clinical studies, however, have reported ambiguities between diagnosis and presentations. The term “KAT6B spectrum disorder” has been proposed to encompass both GPS and SBBYSS (Gannon et al., 2015; Lundsgaard et al., 2017; Radvanszky et al., 2017).

The mutations associated with GPS and SBBYSS largely occur at distinct positions on the *KAT6B* gene, and for classification purposes the *KAT6B* mutations can be divided into four groups (Campeau et al., 2012; Sheikh and Akhtar, 2019). Group 1 mutations promote an SBBYSS phenotype and cluster within exons 15, 16, and in the proximal region of 17 of the *KAT6B* gene (although rare cases with early exon mutations also exist in this group) (Vlckova et al., 2015; Lundsgaard et al., 2017; Radvanszky et al., 2017). Group 2 mutations, which promote GPS phenotypes, are located in distal exon 17 and the proximal portion of exon 18 (Vlckova et al., 2015; Radvanszky et al., 2017). Group 3 mutations occur in the medial portion of exon 18, and give rise to GPS, SBBYSS, and mixed GPS/SBBYSS phenotypes (Vlckova et al., 2015; Radvanszky et al., 2017). Group 4 mutations are found in the distal section of exon 18 that encodes for the transactivation domain of KAT6B, and are associated with SBBYSS phenotypes (Vlckova et al., 2015; Radvanszky et al., 2017). The majority of mutations that cause both the GPS and SBBYSS clinical presentations are non-sense or frameshift, although some missense mutations and in-frame deletions have also been identified (Gannon et al., 2015; Lundsgaard et al., 2017). The postulated mechanism of pathogenesis is haploinsufficiency or loss-of-function for SBBYSS, and gain-of-function or a dominant-negative mechanisms for GPS (Campeau et al., 2012; Gannon et al., 2015). This would account for the milder phenotypes generally associated with SBBYSS (Campeau et al., 2012; Gannon et al., 2015). However, direct experimental evidence to support this assertion is lacking to date. Normally, KAT6B can integrate into complexes that regulate transcription, and is known to also partner with RUNX2 and form a tetrameric complex with BRPF1/2/3, hEAF6, and ING5 (Yang, 2015). In mice, *KAT6B* expression in the brain is dynamic during early development, after which it remains highly expressed in neural stem cells in the subventricular zone throughout adulthood (Thomas et al., 2000; Sheikh et al., 2012). KAT6B predominantly acetylates H3K9 and H3K23 and is critical for maintaining the multipotent and self-renewing properties of neural stem cells (Campeau et al., 2012; Sheikh et al., 2012; Sheikh and Akhtar, 2019).

There have been few animal models of KAT6B deficiency generated to date. Thomas et al. using random gene trap methodology generated a mouse model in which only 10% of the normal mRNA expression of Querkopf (*Qkf*), the mouse ortholog of *KAT6B*, was observed (Thomas et al., 2000; Thomas and Voss, 2004). Mice homozygous for the *Qkf* gene trap displayed craniofacial dysmorphisms, decreased body mass, and failure to thrive, with two-thirds not surviving to weaning (Thomas et al., 2000; Thomas and Voss, 2004). The cortex and olfactory bulb of these mice were smaller than normal, possessing fewer cells overall, GAD67-positive interneurons and large pyramidal neurons in the cortex (Thomas et al., 2000). Impairments in adult neurogenesis have also been identified in these mice, with reduced numbers of neural stem cells in the subventricular zone and granule and periglomerular interneurons in the olfactory bulb (Merson et al., 2006).

### Conditions Associated With Impaired Histone Deacetylase Activity

#### Brachydactyly Mental Retardation Syndrome

Brachydactyly mental retardation syndrome (BDMR; OMIM #600430) is a developmental condition that shares a number of similar clinical features with Albright hereditary osteodystrophy (Leroy et al., 2013; Wheeler et al., 2014). Cognitive impairment and developmental delay were originally thought to be universal, although closer evaluations suggest developmental delay is clearly evident in ~79% of patients (Wheeler et al., 2014; Le et al., 2019). Autistic-like behaviors are also a common feature in BDMR (Williams et al., 2010). Additional neurological impairments, such as hypotonia and seizures, are present in ~46% of patients (Williams et al., 2010; Le et al., 2019). In addition, brachydactyly type E was observed in approximately half of patients, with the fourth and fifth metacarpal being the most severely affected (Le et al., 2019). Other features observed include distinctive craniofacial dysmorphisms, obesity, sleep disturbances, and cardiac defects (Morris et al., 2012; Le et al., 2019).

BDMR is associated with variably sized deletions of the telomeric region of chromosome 2q37. The boundaries of the commonly deleted region encompass over 197 genes and the identity of the primary causal gene(s) for the condition was initially unclear (Leroy et al., 2013). However, deletion mapping identified *HDAC4* as a common deleted gene in most patients, including those with the smaller microdeletions of 2q37.3 (Villavicencio-Lorini et al., 2013; Le et al., 2019). Moreover, a recent report identified a BDMR patient whose causal mutation was a one base insertion into the HDAC4 gene open reading frame (Williams et al., 2010). Collectively, these observations implicate *HDAC4* as critical gene in BDMR, and suggest that HDAC4 haploinsufficiency represents a pathogenic mechanism for BDMR (Williams et al., 2010; Wheeler et al., 2014). Further support for this possibility comes from genotype-phenotype studies, where common features seen in BDMR patients that include skeletal development, obesity, and behavioral tendencies have been reported (Leroy et al., 2013).

HDAC4 is a Class IIa HDAC, and like all members of this subfamily, contains a specific alteration - a His976Tyr in its catalytic domain (Mielcarek et al., 2015). This alteration abolishes the deacetylase activity of HDAC4, and thus its effects on histone acetylation are exerted through its transcription binding domain (TBD), allowing it to form specific complexes to repress gene expression (Williams et al., 2010; Mielcarek et al., 2015). Mutations in *HDAC4* alter its ability to recruit required enzymes. Notably, it has been postulated that HDAC4 can act as a scaffold for the N-CoR/HDAC3 complex and enhance HDAC3-mediated H3 and H4 lysine deacetylation, at H4K5, K8, K12, and K16 (McQuown and Wood, 2011; Seto and Yoshida, 2014; Mielcarek et al., 2015). HDAC3/4 complexes will also deacetylate H2A at K5 (Seto and Yoshida, 2014). An *Hdac4*-null mouse, however, showed no change in the acetylation status of histones at age P3 (Mielcarek et al., 2013). Examination of histone acetylation in older *Hdac4*-null mice have yet to be reported and could prove informative.

*Hdac4* is highly expressed in the brain, with expression levels peaking in early postnatal life during periods of heightened synaptogenesis (Sando et al., 2012; Mielcarek et al., 2013). The importance of Hdac4 during this stage of development was investigated using mice, in which an inserted lacZ cassette caused the deletion of the TBD, nuclear localization signal, and catalytic domains of the encoded protein (Vega et al., 2004). These mice were severely undersized, presented skeletal defects, and did not survive to weaning (Vega et al., 2004). A separate study found the brains of *Hdac4* knockout mice were 40% smaller than wild-types, and had enlarged ventricles (Majdzadeh et al., 2008). Progressive degeneration of Purkinje neurons was also noted P3 to P10, with surviving neurons displaying stunted dendrite growth (Majdzadeh et al., 2008). It is worth noting, though, that *Thy1-Cre/Hdac4* and *Nes-Cre/Hdac4* conditional knockout mice have normal locomotor activity and display normal brain morphology (Price et al., 2013). Both of these transgenic lines ablated *Hdac4* from large populations of neurons, thereby raising the possibility that Hdac4 impairments could promote effects in neurons through non-cell autonomous mechanisms. However, when Hdac4 was absent in forebrain excitatory neurons in another conditional knockout mouse model, *CamKII-Cre/Hdac4*, the mice were hyperactive, with deficits in memory and motor coordination, and reduced anxiety-like behaviors (Kim et al., 2012). Furthermore, a transgenic mouse model harboring truncated Hdac4 had attenuated excitatory synaptic strength, and impaired spatial memory (Sando et al., 2012). Taken together, these studies illustrate that Hdac4 deficiency alone is sufficient to induce neurological and peripheral consequences that have commonality with BDMR patients. Despite these data, the potential involvement of other genes deleted on chromosome 2q37 in some patients cannot be ruled out for a potential contributing role in pathogenesis.

## Discussion

**Figure 3:**
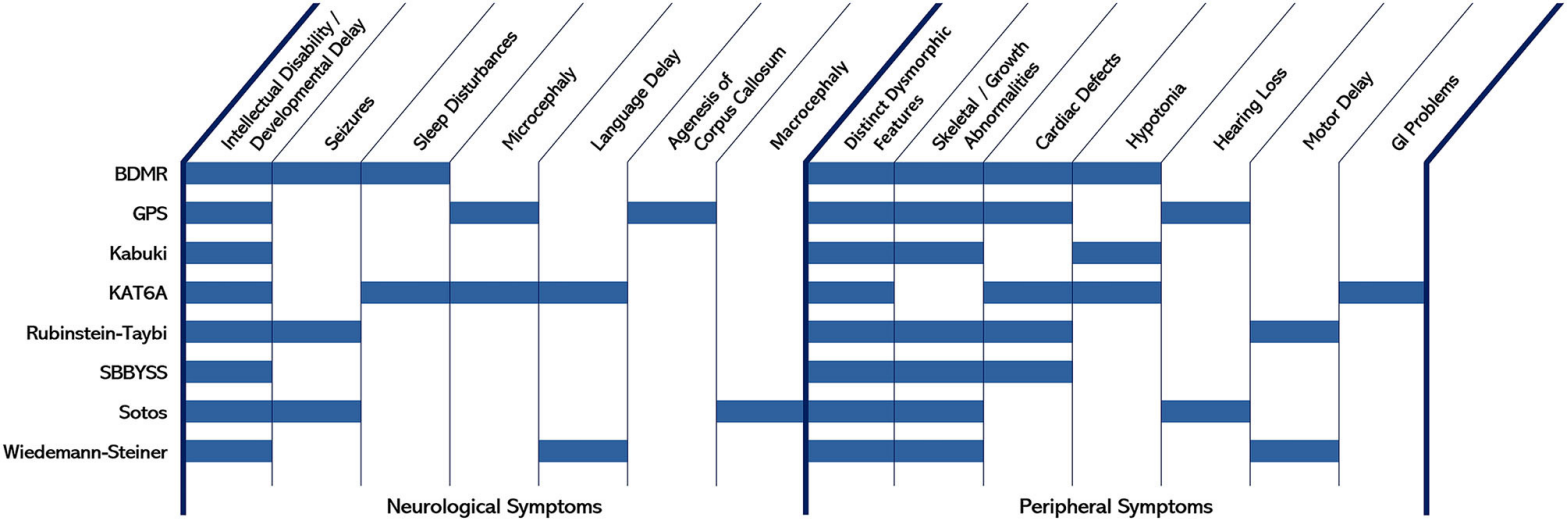
Neurological and peripheral symptoms common to different epigenetic-linked developmental conditions.

Epigenetic modifications, and the functional fidelity of systems directly involved in making these modifications, clearly play important roles in neurodevelopment. Epigenetic modifications of histones specifically, whether through methylation or acetylation, can have affects on individual gene regulation or effects on multiple genes, since large regions of chromatin can be influenced by these histone modifications. Inappropriate regulation can lead to impairments in constitutive or cue-dependent activation or deactivation of genes. Indeed, model systems investigating specific enzymes involved in coordinated histone methylation or acetylation have revealed complete ablation of the enzyme frequently results in embryonic lethality, with defects in germ layer formation and/or gross impairments in early developmental ontogenesis often observed. In haploinsufficient or functionally-hypomorphic systems, alterations in the normal homeostatic activity range for the pathways encoded by these factors can be sufficient to cause severe conditions that manifest in early childhood. For a number of these cases, similar or overlapping clinical features can be observed even though the specific gene mutated may encode a factor that seemingly regulates opposite aspects of epigenetic control. Indeed, mutations affecting several enzymes and involving multiple systems can result in widespread consequences that collectively impair neurological, skeletal, and cardiac development (amongst others) from properly occurring. Although the expression profiles of these enzymes vary, the convergence of phenotypes that affect the central nervous system and peripheral systems is clear (Figure 3). Development requires complex but orchestrated spatiotemporal changes in gene expression, which is strongly mediated by cues provided through histone modifications. If the fidelity of this mechanism is impaired, the normal orchestrated response fails, cue-dependent developmental patterning does not properly execute, and the ontogeny of normal synaptic and network formation is negatively impacted in the developing brain.

The effect of histone methylation and acetylation on general chromatin structure and gene expression is well-documented (Bannister and Kouzarides, 2011). It is also important to note, however, that the specific enzymes catalyzing these histone modifications may also possess dual or multi-modal functionality and regulate other non-histone systems. Certain tumor suppressor factors have been found to be acetylated by various HATs; for instance, both CBP and p300 target p53, p73 E2F, and Rb to affect their respective binding to promoter regions (Sterner and Berger, 2000; Iyer et al., 2004). PCAF shares many of these same targets, and KAT6A also is known to acetylate p53 (Sterner and Berger, 2000). These modifications can influence transcriptional responses in conjunction with, or independently of, MOZ-dependent histone acetylation (Huang et al., 2016). Thus, while mutations of histone acetylation or methylation factors alter normal epigenetic histone coding, other targets may also be affected whose functions may extend to systems not classically viewed as “epigenetic.” Likewise, gene regulation within different regions of the genome that are subject to the same modification may not respond equally. While histone modifications affect chromatin structure and enable/restrict transcriptional responsiveness, the actions of specific transcription factors and transcriptional complexes dictate the dynamics of the response. Moreover, the activity of one histone modification system can alter specific histones in a manner that better enables the recruitment of additional systems. For example, the methylation of H3K4 by KMT2 family members allows for a higher-affinity recruitment of MYST acetyl-transferases to those sites. The ensuing acetylation of the histone H3 is associated with increased local transcriptional activation (Sheikh and Akhtar, 2019). If the initial histone modification is impaired, the subsequent recruitment that directly effects transcription will not properly occur. Thus, altering the activity of one histone modifier can have broad impact on a multitude of transcriptional regulatory complexes that normally function in a cooperative manner to achieve a proper homeostatic response. In addition, several histone methyltransferase and acetyltransferase proteins include motifs that bind other transcriptional regulators to allow complexes to assemble. In many cases, these additional motifs reside downstream of the catalytic motif in the expressed transcript. Since they reside distally, these domains would also be affected by mutations that induce nonsense-mediated decay. Thus, not only is the catalytic activity of the transferase lost, but critical interactions dictated by these more distal motifs are also compromised. This is especially important for Class IIa HDACs, for instance HDAC4, which do not have intrinsic deacetylase activity, but regulate gene expression through the complexes they form with other transcription factors.

In summary, epigenetic processes conveyed by post-translational modifications of histone proteins represent a key component in the overall regulation of static and dynamic gene expression. The neurodevelopmental disorders discussed in this review highlight the neurological (along with peripheral and/or embryonic development) consequences that can arise from impaired histone acetylation or methylation coding. A failure in normal cue-dependent execution of epigenetic signaling at any of several key developmental windows can lead to the same endpoint, which may explain why common features can emerge in patients affected by different causal mutations. Further research into these post-translational histone modification diseases, and developing additional model systems to complement those currently available, will be important to delineate a clearer understanding of the molecular, cellular and pathological mechanisms underlying these conditions, and ultimately the development of translationally relevant therapeutics.

## Author Contributions

MF: wrote sections of manuscript, generated table, and compiled bibliography. DS: wrote sections of manuscript, generated figures, and proof-read manuscript. CR: generated figures, edited manuscript, and contributed to discussion section. JE: selected topic of review, oversaw completion of text, edited and proof-read manuscript, compiled, and submitted manuscript. All authors contributed to the article and approved the submitted version.

## Conflict of Interest

The authors declare that the research was conducted in the absence of any commercial or financial relationships that could be construed as a potential conflict of interest.
